# Investigation of Play Intervention for Dementia (PID) Activities in Addressing Cognitive Domains Reflected in Hong Kong Montreal Cognitive Assessment (HK-MoCA)

**DOI:** 10.1177/23337214221130161

**Published:** 2022-10-17

**Authors:** Calvin Ka-Fung Lo, Eric Andrew Yung, Ka Tat Tsang

**Affiliations:** 1 Department of Pathology and Laboratory Medicine, University of British Columbia, Vancouver, Canada; 2 School of Public Health Sciences, University of Waterloo, ON, Canada; 3 Factor-Inwentash Faculty of Social Work, University of Toronto, ON, Canada

**Keywords:** Strategies & Skills Learning & Development, Alzheimer’s disease, mild cognitive impairment, dementia, long-term care

## Abstract

**Background/Objectives:** Yee Hong Play Intervention for Dementia (PID) is a community program strengthening East Asians >65 years with dementia in their daily functional activities. We analyzed how PID activities align with Hong Kong Montreal Cognitive Assessment. **Methods:** Utilizing observation sheets procured from documentation notes from the twice-weekly PID sessions, cognitive domains were identified. Mean time duration and activity frequencies were compared between high and low competency client groups. **Results:** Independent of competency group, activities predominantly targeted attention/concentration (23.8% HC, 16.4% LC), and hand-eye coordination (19.1% HC, 28.7% LC). Less focused domains were delayed recall (3.1–4.7%) and naming (1.3–1.5%). **Conclusions:** Yee Hong PID tested innovative cognitive domains not currently covered in HK-MoCA screening assessment, emphasizing attention/concentration-oriented activities and none assessing orientation and language domains. Additionally, presence of new domains such as hand-eye coordination and fine motor dexterity suggested that strict adherence with standardized screening tools (e.g., MoCA) may not be ideal. Likely, facilitators have developed innovative measures to assess individual competency to strengthen resilience in our geriatric population.

## Introduction

Cognitive assessments are typically conducted to evaluate and diagnose individuals who may be at risk or are currently affected by cognitive impairment (Gluhm et al., 2013; Yeung et al., 2014). Mild cognitive impairment (MCI) is a transition stage associated with early-stage dementia, a disorder that typically impairs cognitive function in memory domain with age (Yeung et al., 2014). Depending on diagnostic criteria, prevalence of MCI in individuals over 65 years of age ranges from 3 to 13% (Gluhm et al., 2013; Yeung et al., 2014). The most widely utilized tests to assess for impairment include a) Mini-Mental State Examination (MMSE) and b) Montreal Cognitive Assessment (MoCA). However, MoCA has been suggested to more effectively measure cognitive performance when taking into account repeated measures in decadal age increments (Gluhm et al., 2013; Yeung et al., 2014), education (Wang et al., 2013), ceiling effects (Trzepacz et al., 2015) (MoCA 18% vs. MMSE 71%), and higher sensitivity in detecting MCI (Damian et al., 2011; Ismail et al., 2010; Montiel et al., 2013; Pan et al., 2012). This in turn has led to greater reliance on MoCA and its variants (i.e., Hong Kong MoCA or HK-MoCA) which mitigates aforementioned issues within MMSE while effectively differentiating between healthy and MCI clients with greater sensitivity.

Despite confidence in using standard screening tools, the aforementioned studies put particular emphasis on comparison studies and test validation in conducting MoCA. New assessment tools are continuously suggested to accommodate and enhance detection of MCI, including Hong Kong Brief Cognitive Test (HKBC) to supplement HK-MoCA (Chiu et al., 2018). Besides domains represented in HK-MoCA, additional domains such as “General Knowledge” (i.e., name of current leader or country) and “Frontal lobe function” (hand motion imitation) were implemented within HKBC (Chiu et al., 2018). Despite implementation of these screening tools in clinical assessments for patients with suspected cognitive impairment, there remains limited studies in terms of application of such objective tools within geriatric activity programs, including those with community-based outreach programs.

PID (Play Intervention for Dementia) (Tsang, 2017) is a program formulated under the SSLD (Strategies & Skills Learning & Development) System (Tsang, 2013) which supplements HK-MoCA. This program aims to encourage Persons with Dementia (PWD) > 65 years to have enjoyment in daily living and reinforce their participation in their activities of daily living (ADL) and instrumental activities of daily living (iADLs), create interpersonal relationships through various forms of interaction, and enhance cognitive capacities (Tsang, 2018). PID sessions typically include splitting clientele into high and low competency groups. Instead of objective classifications (e.g., MMSE or MoCA score cutoff values), clients were designated into either competency group based on their physical limitations and extent of assistance with volunteer staff (refer to *Methods*). Each group is then presented a variety of simulated activities that combine physical toys, modified game sets and miscellaneous materials (i.e., paper, sticks, containers) which PID-trained staff and volunteers creatively design. As per PID guidelines, the time limit for each activity is 8 minutes to maximize client engagement before moving to the next activity (Kopecek et al., 2017). Their main goal is to measure different cognitive domains associated with dementia, including but not limited to fine sensory-motor coordination, engagement, and communication. Since its development in 2013, clinical studies addressing effectiveness and feasibility of PID revealed program satisfaction from all participating clients (Cheung et al., 2019; Li & Ho, 2019). Additionally, results revealed a significant difference (*p* = .028) in MoCA scores specifically in the memory domain between geriatric clients in PID-treated group versus their control group (no PID treatment) (Cheung et al., 2019). Further suggestions included the use of video elicitation focus group interviews (individual counseling sessions) and participatory videos (PWD and staff collaborative community films) to effectively communicate the needs of PWD with dementia to practitioners and social workers (Li & Ho, 2019). However, questions remain on effectiveness of PID activity distribution and their ability to assess domains reflected in MoCA in support of geriatric communities with various stages of dementia.

HK-MoCA will be our cognitive screening tool of focus given our predominantly Chinese and Oriental Asian clientele at Yee Hong. Over a 11-month period (January 4 to December 6, 2019), our study objective analyzed how current PID activities align with standard screening tools such as HK-MoCA with respect to their cognitive domains, and further explore any recurring domains reflected in PID activities that were less thoroughly addressed in HK-MoCA. Additionally, we believe that specific cognitive domains may be more significant when assessing populations marked by differences in cultural and ethnic backgrounds (Shim et al., 2017; Zhai et al., 2016). Additional studies have suggested splitting MoCA normative data based on culture-dependent education (due to cultural differences resulting in falsely low test scores) (Apolinario et al., 2018; Carson et al., 2018; Ihle-Hansen et al., 2017; Mellor et al., 2016; Zhou et al., 2015) and age (older clients resulting in lower scores) (Borland et al., 2017; Kopecek et al., 2017; Krist et al., 2019; Rossetti et al., 2017; Santangelo et al., 2015; Thomann et al., 2018) to raise test sensitivity and specificity pertaining to their non-Western target area demographic. In conclusion, we hope our findings will help address any gaps or differences in the application of MoCA in a clinical setting in comparison to community outreach programs. Given our predominant East Asian and Southeast Asian clientele, in the clinical setting, cultural barriers may exist where clients may withhold information from health care providers thus affecting diagnosis or treatment options. Consulting with community-based programs that cater to this cohort can present opportunities for confiding of personal and medical challenges, creating a multidisciplinary approach to address client needs who immigrate to Canada. Identifying these insights can promote a more effective method and future programs that can differentiate the needs of PWD based on cultural background, and ethnicity.

Our research question is as follows: Do PID activities at Yee Hong Centre for Geriatric Care (Mississauga) address the cognitive domains reflected in HK-MoCA? We are conducting a retrospective evaluation of program activities, comparing their assessed domains to HK-MoCA (which would be applied in a standardized clinical setting). Our goal is to identify if significant differences exist between the characteristics of Yee Hong PID activities conducted for the “High Competency” compared to the “Low Competency” client groups, whether it between frequency of domains, or duration per domain-specific activity.

## Methods

Yee Hong Centre for Geriatric Care (Mississauga) is a non-profit long-term care facility which provides a spectrum of services ranging from community-based recreational activities for clients age 50+ (e.g., Tai Chi, Yoga, congregate dining), with more focus toward intervention groups in the form of Adult Day Program, to housing services and long-term care.

Within each PID session, there are two client groups subjectively differentiated by the PID Program Facilitators as: a) High Competency (HC) and b) Low Competency (LC) to create approximately similar clientele in terms of cognitive functioning. Competency was defined as a surrogate measure, based on extent of assistance (i.e., low competency clients require client: volunteer ratio of 2:1; high competency clients require client: volunteer ratio of 3:1 or greater), and overall mobility including use of wheelchairs.

Separate assessments of data were conducted for each competency group. Furthermore, this observational study did not include direct identifiers or performance of individual clients because as mentioned previously, the aim is to strictly assess the cognitive domains addressed in PID activities and their respective time durations rather than assessing individual client performance. Specifically, client engagement was measured as amount of time spent per activity or number of clients attending this activity.

This protocol was reviewed under the University of Toronto Health Sciences Research Ethics Board (REB) and approved on July 5th, 2019. The collection period lasted from January 4 to December 6, 2019. Four key components were analyzed in detail from these observation sheets:

1) Assessed Cognitive Domain in each Activity
2) Duration (minutes) of each Activity
3) Outstanding Notes on Effectiveness of Activities (e.g., client engagement, observed difficulties with certain activities, and skipped activities)

Statistical analysis was conducted using software tools including Excel and RStudio (V 1.2.5001, Boston, Massachusetts). Session activities were aligned to a set of cognitive domains reflected in HK-MoCA. These domains include: Visuo-constructional Skills (VS), Naming (N) or the ability to refer to an object, person, place, concept or idea by its proper name, Memory (M), Attention & Concentration (AC), Language (L), Abstraction (A), Delayed Recall (DR), and Orientation (O), along with unique domains not reflected in HK-MoCA including Creativity (C), Fine Motor Dexterity (FM), and Hand Eye coordination (HE).

Two-sample *t*-tests were performed to measure differences between mean duration of each activity irrespective of domain between HC and LC groups. Follow-up two-sample *t*-test analyses were then conducted for each cognitive domain to measure differences in mean duration between HC and LC group activity. Secondary analysis included separate one-way ANOVAs on each competency group to compare mean time durations per domain, followed by two-way ANOVA to measure significant differences in mean time duration per domain across groups.

To assess duration, single-sample *t*-tests were performed to compare mean duration per domain in both groups to the null hypothesis represented by a recommended SSLD standard of 8 minutes per activity (Kopecek et al., 2017).

Finally, separate χ^2^ analyses were conducted for each competency group to compare frequency of each observed domain (both novel and those reflected in HK-MoCA) to an expected distribution (i.e., null assumes that all domain will be covered equally).

In summary, we interpret our findings to align with the following objectives:

a) Which cognitive domains were covered most and least extensively at the end of collection period?
b) Any domains presented in PID that are not specifically reflected in HK-MoCA?
c) Noticeable patterns that affect client engagement to staff/volunteer-led activities? (e.g., icebreakers, naming introductions, complexity of activities, connection to ADLS)?
d) Variances in instruction or performance time across specific domains (and do these durations change between competency groups)?
e) Does mean duration of each domain-associated activity follow recommended guidelines listed in PID resource guides?

## Results

A total of 85 sessions (January 4 to December 6, 2019) documenting 1424 activities (Table 1) and their associated domains and durations were analyzed. However, approximately 40% of the data harbored missing values (predominantly in the first 4 months of sampling) and therefore sample size was adjusted accordingly for each statistical analysis conducted.

**Table 1.** Mean Number Activities Covered Per Competency Group Per Session.

Competency group	# Activities covered/session	Total # activities (*n*)
HC	9.26	787
LC	7.49	637

*Note.* Total sample size of 85 sessions were transcribed and analyzed with *n* = 787 and 637 for high and low competency groups respectively.

Through two-sample *t*-tests, mean activity durations were compared between HC and LC groups. PID staff and volunteers did not follow the 8 minutes per activity standard and exceeded these limits for both HC and LC groups (12.02 and 11.05 minutes, respectively) (Chiu et al., 2018), significant time differences also existed between both groups (mean difference [md] = +0.97 minutes for HC, *p* = .0417). Please refer to Table 2 for individual values. Additional two-sample *t-*tests between HC and LC groups (Table 3) yielded no significant differences when comparing for each domain.

**Table 2.** Average Duration (Minutes) Spent Per Activity for High and Low Competency Groups Over an 11-Month Session (January 4 to December 6, 2019) Followed by Two-Sample *t*-Test to Measure for Significant Differences.

Competency group	Average duration (minutes)/activity
HC	12.02
LC	11.05
Mean difference (minutes)	0.97
*p*-Value	.0417*
Effect size (Cohen’s *D*)	0.17
Degrees of freedom	560.89
95% CI	0.037–1.90

*Note*. p-Values marked with an * denote significant differences. Significant values are accompanied with their corresponding effect sizes using Cohen’s D.

**Table 3.** Mean Duration Spent Per Domain Across Low and High Competency Groups.

Average duration (minutes) per domain/group	A	AC	C	DR	FM	HE	M	N	VS
HC	13.833 (24)	12.500 (46)	13.923 (13)	9.857 (7)	11.667 (33)	10.054 (37)	12.062 (16)	N/A	14.889 (9)
LC	12.071 (12)	11.750 (40)	12.350 (20)	7.600 (10)	11.125 (32)	10.343 (70)	11.577 (26)	N/A	9.357 (14)
Mean difference (minutes)	1.762	0.75	1.573	2.257	0.542	0.289	0.485	N/A	5.532
*p*-Value	.389	.574	.533	.232	.697	.748	.787	N/A	.0929
95% CI	−2.36 to +5.89	−1.89 to +3.39	−3.52 to +6.67	−1.62 to +6.13	−2.23 to +3.31	−2.07 to +1.50	−3.16 to +4.13	N/A	−1.09 to +12.16

*Note*. Two-sample *t*-tests were individually conducted for each domain from both competency groups. “Naming” column was denoted “NA” due to small sample size (<5). Associated brackets adjacent to HC and LC mean values represent their sample sizes.

Separate one-way ANOVAs measuring mean duration across domains in HC and LC groups (Table 4) yielded no significant findings. Two-way ANOVA comparing duration between both groups resulted in a significant difference for the domain variable (*p* = .039). However, post-hoc tests (Table 5) revealed none of the comparisons (i.e., identical domains between HC and LC) to be significantly different. Mean duration for “Naming” domain was omitted due to low occurrence rate (i.e., *n* < 5) in observation sheets.

**Table 4.** One-Way ANOVA Analyses Between Mean Duration per Domain Within High and Low Competency Groups.

Group	Statistic	*p*-Value
HC	One-way ANOVA (within HC group)	.128
LC	One-way ANOVA (within LC group)	.340
LC/HC	Two-way ANOVA (comparison between groups)	Domain—.0388* (η^2^ = 0.0388)
		Group—.0952
		Domain: Group—.677

*Note*. Additional two-way ANOVA conducted with independent variables: Domain and Group, and response variable Duration. p-Values marked with an * denote significant differences. Eta squared (η^2^) was calculated to analyze effect size of domain variable only.

**Table 5.** Two-Way ANOVA Follow-Up Post-Hoc Tests Between Identical Domains Between Competency Groups.

Pairwise comparison	Comparison *p*-value
A:LC-A:HC	0.9999758
AC:LC-AC:HC	1.0000000
C:LC-C:HC	0.9999980
DR:LC-DR:HC	0.9999966
FM:LC-FM:HC	1.0000000
HE:LC-HE:HC	1.0000000
M:LC-M:HC	1.0000000
N:LC-N:HC	1.0000000
VS:LC-VS:HC	0.6717222

**Figure 1.**
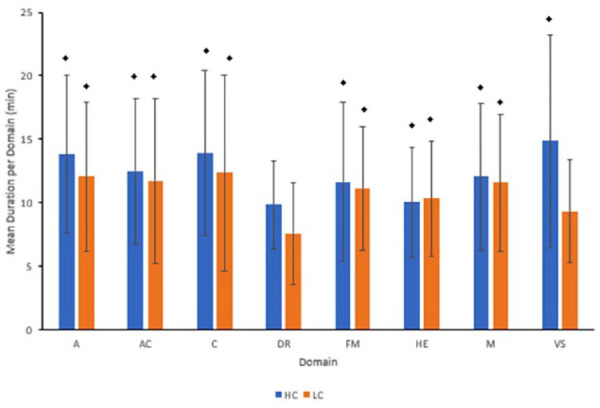
Depiction of mean duration (minutes) per domain under high (left bars) and low (right bars) competency groups with ± standard error bars. A diamond symbol denotes a significant difference in mean duration from the expected PID standard of 8 minutes.

Single-sample t-tests were conducted comparing each mean duration from both LC and HC groups to 8 minutes (null) as observed in Table 6 and Figure 1. Due to lack of occurrences, “Language,” “Orientation,” and “Naming” domains were omitted. Under HC group, mean duration for “Abstraction” (md = +5.833 minutes, *p* < .0001), “Attention/Concentration” (md = +4.5, *p* < .0001), “Creativity” (md = +5.923, *p* < .01), “Fine Motor Dexterity” (md = +3.667, *p* < .01), “Hand-eye Coordination” (md = +2.054, *p* < .01), “Memory” (md = +4.062, *p* = .013), and “Visuospatial” (md = +6.889, *p* = .039) were significantly longer than the recommended 8-minute limit. Under LC group, domains significantly longer than the recommended limit included “Abstraction” (md = +4.071, *p* = .022), “Attention/Concentration” (md = +3.75, *p* < .001), “Creativity” (md = +4.35, *p* = .021), “Fine Motor Dexterity” (md = +3.125, *p* < .001), “Hand-Eye Coordination” (md = +2.343, *p* < .0001), and “Memory” (md = +3.577, *p* = .0024).

**Table 6.** Average Duration (Minutes) Spent per Domain for Both Competency Groups Compared to Recommended Duration of 8 Minutes Through Single-Sample *t*-Test Analysis.

Average time (minutes)/domain (*p*-value)/competency group	A	AC	C	DR	L	O	FM	HE	M	N	VS
HC	13.833 (24)	12.500 (46)	13.923 (13)	9.857 (7)	N/A	N/A	11.667 (33)	10.054 (37)	12.062 (16)	N/A	14.889 (9)
HC: difference from 8 minute standard (minutes)	5.833	4.500	5.923	1.857	N/A	N/A	3.667	2.054	4.062	N/A	6.889
95% CI	11.21–16.46	10.81–14.19	10.01–17.83	6.68–13.03	N/A	N/A	9.45–13.88	8.60–11.50	8.99–15.13	N/A	8.45–21.32
*p*-Value (effect size)	<.0001* (*d* = 0.94)	<.0001* (*d* = 0.79)	<.01* (*d* = 0.91)	0.207	N/A	N/A	<.01* (*d* = 0.59)	<.01* (*d* = 0.47)	.013* (*d* = 0.70)	N/A	.039* (*d* = 0.82)
LC	12.071 (12)	11.750 (40)	12.350 (20)	7.600 (10)	N/A	N/A	11.125 (32)	10.343 (70)	11.577 (26)	N/A	9.357 (14)
LC: difference from 8 minute standard (minutes)	4.071	3.75	4.35	-0.4	N/A	N/A	3.125	2.343	3.577	N/A	1.357
95% CI	8.69–15.46	9.67–13.83	8.74–15.96	4.75–10.44	N/A	N/A	9.38–12.87	9.26–11.42	9.40–13.75	N/A	7.04–11.67
*p*-Value (effect size)	.022* (*d* = 0.69)	<.001* (*d* = 0.58)	.021* (*d* = 0.56)	0.76	N/A	N/A	<.001* (*d* = 0.64)	<.0001* (*d* = 0.52)	.0024* (*d* = 0.66)	N/A	0.23

*Note*. Corresponding brackets adjacent to each mean represent samples size. *p*-Values marked with an * denote significant differences. Significant values are accompanied with their corresponding effect sizes using Cohen’s *D*.

χ^2^ Test of independence was conducted and showed significant association between competency group and their corresponding frequency domain count (χ^2^ = 25.419, *p* = .001, Tables 7 and 8). χ^2^ Goodness of fit tests were subsequently conducted on pretense of an expected null distribution (assuming all nine domains analyzed below are equally distributed). The result was a significant difference from expected for both HC (*p* = .0013) and LC (*p* < .0001) groups.

**Table 7.** Two-Way Contingency Table Presenting Frequency of Each Domain (Count) Per Competency Group.

Group/competency	A	AC	C	DR	FM	HE	M	N	VS	Total
HC	58	92	32	12	60	74	29	5	25	387
LC	32	67	37	19	60	117	43	6	27	408
Total	90	159	69	31	120	191	72	11	52	795

*Note*. A total of nine domains were tested. Language and Orientation domains (HK-MoCA) were omitted as a result of low sample size (<5). Sample size (*n* = 393) adjusted to omit activities with missing information.

**Table 8.** χ^2^ Test of Independence and Goodness of Fit Analyzed From Group/Competency Contingency Table (Table 7).

χ^2^ Test	χ^2^	*p*-Value	Effect size (Cramer’s *V*)
Test of independence	25.42	.00132*	0.032
Goodness of fit—HC subset only (*df* = 8)	87.69	1.37 × 10^−15^*	0.34
Goodness of fit—LC subset only (*df* = 8)	86.36	2.54 × 10^−15^*	0.33

*Note*. The following descriptions were calculated: χ^2^ statistic, resulting *p*-value, and subsequent effect size represented by Cramer’s *V*. Test of independence compared domains with group type. Separate goodness of fit tests were performed on HC and LC competency groups to fit their observed counts to an expected equal distribution of all nine analyzed domains (1/9). p-Values marked with an * denote significant differences.

Multiple post-hoc binomial test comparisons were subsequently conducted with Bonferroni’s correction, to adjust *p*-values (Table 9) between each domain count and their expected distribution (1/9 of all activities). Significant differences in the following binomial comparisons within HC group were as follows: Attention/Concentration 23.77% (92/387, *p* < .0001), Delayed Recall 3.1% (12/387, *p* < .0001), Hand Eye Coordination 19.12% (74/387, *p* < .0001), Naming 1.29% (5/387, *p* < .0001), and Visuospatial 6.46% (25/387, *p* = .024). Post-hoc binomial tests for LC group revealed significant differences in: Attention/Concentration 16.42% (67/408, *p* = .0106), Delayed Recall 4.66% (19/408, *p* < .0001), Hand Eye Coordination 28.68% (117/408, *p* < .0001), Naming 1.47% (6/408, *p* < .0001), and Visuospatial 6.62% (24/408, *p* = .0241).

**Table 9.** Post-Hoc Binomial Test (HC and LC Groups Respectively) for Each Domain Count to Their Expected Distribution of 1/9.

Group/competency	A	AC	C	DR	FM	HE	M	N	VS
HC	0.169	<0.0001*	0.678	<0.0001*	0.0842	<0.0001*	0.208	<0.0001*	0.0238*
LC	0.301	0.0106*	1.00	<0.0001*	0.243	<0.0001*	1.00	<0.0001*	0.0241*

*Note. p*-Values were adjusted using Bonferroni’s correction method to compensate for multiple comparisons.

* A significant difference between the observed count and their expected count A total of 9 comparisons were made for each competency group.

## Discussion

Over January to December 2019, two response variables were closely analyzed between HC and LC groups: a) mean duration and b) domain. HC groups spent significantly longer times carrying out each activity (md = +0.97 minutes, *p* = .0417). When comparing mean duration per domain between HC and LC groups, there were no significant differences. When compared to the recommended 8 minutes per activity, 7/8 domains were significantly longer—abstraction (HC +5.83 minutes, LC +4.07); attention or concentration (HC +4.50 minutes, LC +3.75 minutes); creativity (HC +5.92 minutes, LC +4.35 minutes); fine motor dexterity (HC +3.67 minutes, LC +3.12 minutes); hand-eye coordination (HC +2.05 minutes, LC +2.34 minutes); memory (HC +4.06 minutes, LC +3.58 minutes), and visuospatial (HC +6.89 minutes). When compared to their expected distribution (i.e., each domain to be covered equally or 1/9 of the session), frequency of two domains were significantly greater: Attention/Concentration and Hand-Eye domains irrespective of competency group. However, within both groups, Delayed Recall, Naming, and Visuospatial (LC only) were all significantly lower than their expected counts.

Referring to the eight MoCA domains, “orientation” and “language” were omitted from frequency counts (Table 6) given their limited presence over our entire data collection period. Given PID program being conducted in group-based activities, it was evident that these domains could not be fully assessed in each client without peer-to-peer influence which would skew individual evaluations. Further, MoCA domains in “naming” specifically were scarce, only noted in 1.29% (5/387, HC) and 1.47% (6/408, LC) of activities. This was also reflected in “delayed recall” at 3.1% (12/387, HC) and 4.66% (19/408, LC) respectively. As activities were usually recommended to cease after 8 minutes, clients are usually unable to have adequate time to properly assess recalling of past material or activities. Therefore, our hypothesis is that corner facilitators (activity designers) are not familiarized with applying this domain.

HC groups on average, spent more time on each activity than low competency groups. We believe this is the case because clients under milder cognitive impairment have the attention and executive function to be more engaged in more complex activities, including those that require collaborative skills. Indeed, lower MoCA scores are expected in clients with more severe MCI (Chiu et al., 2018; Damian et al., 2011; Gluhm et al., 2013; Ismail et al., 2010; Montiel et al., 2013; Pan et al., 2012; Trzepacz et al., 2015; Wang et al., 2013; Yeung et al., 2014), and the inability for these clients to perform basic tasks in MoCA will in turn, translate to reduced activity engagement and quicker need to transition to a variety of activities to stimulate their participation.

Observing domains with significantly higher mean duration (abstraction, attention/concentration, creativity, fine motor dexterity, hand-eye, and memory), we can attribute these differences to the nature and complexity of the activities. For example, abstraction-related tasks include matching shapes, colors, and card pattern recognition. Naturally, these tasks require a longer time for clients to complete. Furthermore, creativity (e.g., spontaneous building-oriented activities) and hand-eye coordination (e.g., tossing, throwing, rolling toys into designated containers) activities require less cognitive capacities, but provide an avenue for clients to express themselves through what they construct. This results in greater engagement when carrying out creative and hand-eye oriented activities.

The same can be expressed in attention/concentration, hand-eye coordination, and memory domains for the LC group. However, it is more likely that the memory domain is due to LC client inability to effectively perform these tasks. Therefore, contrary to ending the activities early, corner facilitators may find themselves spending excess time to better guide these clients to recalling past memories to help them complete tasks. This adjustment in time can be paralleled to a study suggesting that MoCA threshold cut-offs be lowered to accommodate for clinics specifically addressing clients with memory disorders/impairment (Damian et al., 2011).

Both HC and LC groups predominantly covered Attention/Concentration (23.77% HC, 16.42% LC) and Hand-Eye (19.12% HC, 28.68% LC) domain-related activities the most while Delayed Recall (3.10% HC, 4.66% LC), Naming (1.29% HC, 1.47% LC), and Visuospatial (6.46% HC, 6.62% LC) domains were significantly lower. Due to constant adjustments made in PID to better suit client needs, we believe PID staff and volunteers are intentionally prioritizing “Attention” and “Hand Eye” domain-oriented activities because they elicit greater client engagement and social interaction between clients. Such games include Jenga, and throwing/reactionary games (i.e., slap jack, tossing chips, soft balls). However, there is a trade-off in other important domain-related activities such as “Delayed Recall” and “Naming” which are concurrently covered less. We also note that three additional domains not covered in MoCA (Creativity, Fine Motor Dexterity, Hand Eye) were extensively represented at an equal or higher than expected count (null = 1/9). We believe PID has made these domains known in response to the cultural differences in a program with predominantly East Asian clients (Chiu et al., 2018; Tsang, 2013, 2018). To further elaborate, fine-motor-related activities are more pronounced due to the culture of East Asian clients retaining strong ability in dexterity (e.g., chopsticks, calligraphy) as part of their essential ADLs.

Additionally, our 11-month collection range (January to December 2019) may not exhibit the full spectrum of MoCA domains albeit our study reflected the most recent sessions within the community program. Furthermore, confounding variables such as client satisfaction, and intricate adjustments to activity function which may influence domain counts by PID staff and volunteers cannot be adequately measured.

To comprehensively encompass the various domains within HK-MoCA, it is encouraged that PID observers to extend their evaluations beyond the activities and corresponding domains they assess. For instance, instead of pursuing language-oriented activities which may prove too difficult for low competency clients to engage in, language can be referred to the degree of social interaction and ability to understand basic instructions between PID client and peers and staff. Further, we highly encourage PID corner facilitators to incorporate more item-based (i.e., shapes, animals) tasks and objectives to evaluate client in the “naming” domain. To better encompass the delayed recall domain which may not be practical, we suggest the use of icebreakers to encourage learning of client names or favorite hobbies. In this method, corner facilitators can ask clients to recall each other’s names and hobbies without compromising the facilitator’s activity sessions. We also hope that these new domains covered by PID can shed insight into the need to address cultural differences between clients during cognitive screening processes or programs to encourage proactive lifestyles in our geriatric community.
